# Supplementary material for: A Cold Case of Equine Influenza Disentangled with Nanopore Sequencing
Source: Animals (Basel). 2023 Mar 24;13(7):1153. doi: 10.3390/ani13071153 (PMC10093709; doi:10.3390/ani13071153)

Figure S1. Amino acid sequence alignment of strain ITA/2005/Bari with the HA1 of representative of the various H3N8 lineages and sub-lineages. Strain ZAF/2003/South Africa-4, and USA/2003/Ohio-1 (clade Florida 1). Strain GBR/2007/Richmond-1 and POL/2008/Pulawy-1 (clade Florida 2). Strain GBR/1989/Suffolk (Eurasian lineage) and USA/1994/Kentucky-1 (American lineage). Possible N-linked glycosylation sites are shaded in grey. The positions of amino acids, which have been identified as components of antigenic regions, and their corresponding antigenic regions, are indicated (line under the amino acid letter).

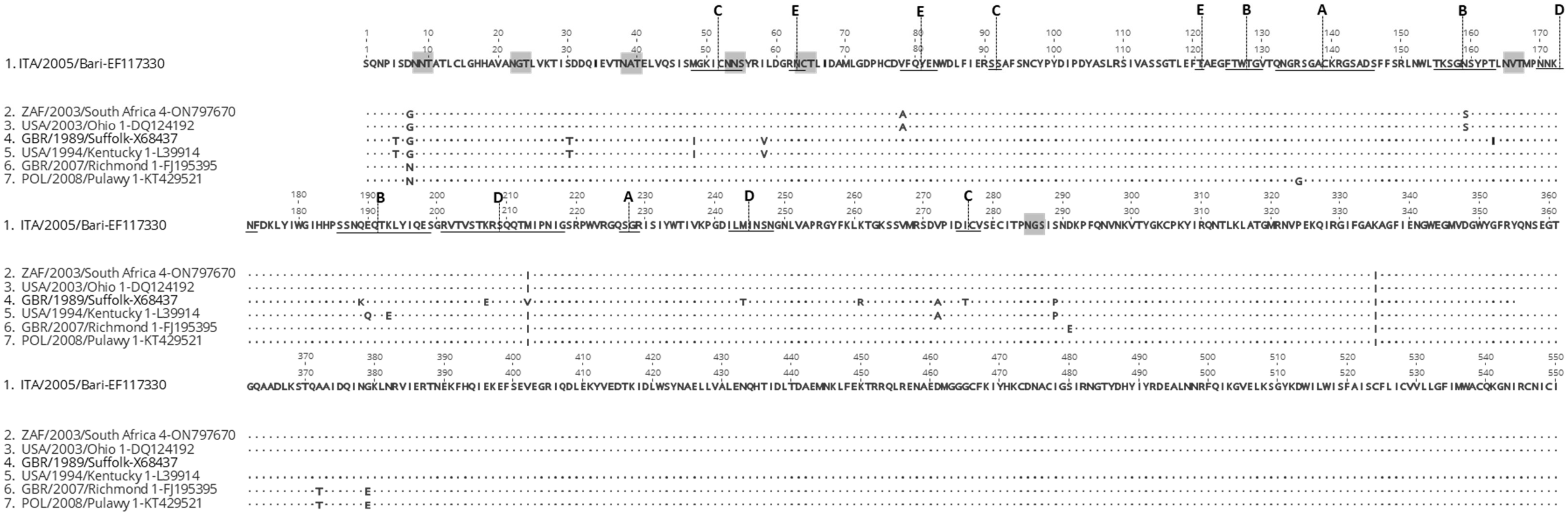

Supplement: Supplementary file 1 [file animals-13-01153-s001.zip › animals-2104690-supplementary.pdf]
